# Identification of the needs and priorities of older people and stakeholders in rural and urban areas of Santo Andre, Brazil

**DOI:** 10.1371/journal.pone.0297489

**Published:** 2024-05-09

**Authors:** Danyela Casadei Donatelli, Dina Goodman-Palmer, Maria Lisa Odland, Sandra Agyapong-Badu, Natalia da Cruz-Alves, Meire Rosenburg, Lisa R. Hirschhorn, Carolyn Greig, Justine Davies, Vânia Barbosa do Nascimento, Eduardo Ferriolli

**Affiliations:** 1 Collective Health Centro Universitário FMABC, Sao Paulo, Brazil; 2 Institute of Applied Health Research, University of Birmingham, Birmingham, United Kingdom; 3 Department of Obstetrics and Gynecology, St Olav’s Hospital, Trondheim University Hospital, Trondheim, Norway; 4 Institute of Life Course and Medical Sciences, University of Liverpool, Liverpool, United Kingdom; 5 Malawi-Liverpool-Wellcome Trust Research Institute, Blantyre, Malawi; 6 Department of Public Health and Nursing, Norwegian University of Science and Technology, Trondheim, Norway; 7 School of Sport, Exercise and Rehabilitation Sciences, University of Birmingham, Birmingham, United Kingdom; 8 Department of Internal Medicine, Ribeirão Preto Medical School, University of São Paulo (USP), Ribeirão Preto, SP, Brazil; 9 Department of Medical Social Sciences and Havey Institute of Global Health, Feinberg School of Medicine, Northwestern University, Evanston, IL, United States of America; 10 MRC-Versus Arthritis Centre for Musculoskeletal Ageing Research, University of Birmingham, Birmingham, United Kingdom; 11 NIHR Birmingham Biomedical Research Centre, University Hospitals Birmingham NHS Foundation Trust and University of Birmingham, Birmingham, United Kingdom; 12 Faculdade de Medicina, Laboratorio de Investigaçao Medica em Envelhecimento (LIM-66), Serviço de Geriatria, Hospital das Clinicas, Disciplina de Geriatria, Universidade de São Paulo, São Paulo, Brazil; FMUP: Universidade do Porto Faculdade de Medicina, PORTUGAL

## Abstract

**Background:**

There are few data reporting the needs and priorities of older adults in Brazil. This hampers the development and/or implementation of policies aimed at older adults to help them age well. The aim of this study was to understand areas of importance, priorities, enablers and obstacles to healthy ageing as identified by older adults and key stakeholders in both urban and rural environments.

**Methods:**

Two locations were selected, one urban and one rural in the municipality of Santo André, in the metropolitan region of São Paulo (SP). Workshops for older adults (>60 y) and stakeholders were conducted separately in each location. The workshops incorporated an iterative process of discussion, prioritisation and ranking of responses, in roundtable groups and in plenary. Areas of commonality and differences between older adult and stakeholder responses were identified by comparing responses between groups as well as mapping obstacles and enablers to healthy ageing identified by older adults, to the priorities identified by stakeholder groups. The socio-ecologic model was used to categorise responses.

**Results:**

There were few shared responses between stakeholders and older adults and little overlap between the top ranked responses of urban and rural groups. With respect to areas of importance, both stakeholder groups ranked policies for older people within their top five reponses. Both older adult groups ranked keeping physically and mentally active, and nurturing spirituality. There was a marked lack of congruence between older adults’ obstacles and enablers to healthy ageing and stakeholder priorities, in both urban and rural settings. Most responses were located within the Society domain of the socio-ecologic model, although older adults also responded within the Individual/ Relationships domains, particularly in ranking areas of most importance for healthy ageing.

**Conclusions:**

Our results highlight substantial differences between older adults and stakeholders with respect to areas of importance, priorities, enablers and obstacles to healthy ageing, and point to the need for more engagement between those in advocacy and policymaking roles and the older people whose needs they serve.

## Introduction

Brazil has undergone a sharp demographic transition, with the number of older people aged ≥60 years increasing from 3 to 20 million between 1960 and 2010 and with a life expectancy in 2023 of 76.2 years [1]. In 2060, it is estimated that older people will represent 32.2% of the Brazilian population, reaching 73.4 million people [2]. Since the Federal Constitution of 1988 established the guarantee of the rights of the aged, there has been a progressive increase in the number of programs and actions dedicated to this population. These include protection of pension payments, free public transport and representative councils for the older people, reaffirmed in the National Policy for Older People, PNI, Law n° 8842, of 1994. The PNI was considered quite advanced for the time, but has not yet been fully applied [3,4].

With regard to health, the main objective of the more recent National Health Policy for Older People (PNSI), issued in 2006, is to promote healthy ageing. This means preserving functional capacity, autonomy and maintaining the quality of life of older people, in line with the principles and guidelines of the Unified Health System (SUS), which offers free access to health services [3,5].

Knowledge of the experiences and perceptions of older people and those who care for them, with respect to the priorities, resources, facilitators and barriers for the promotion of healthy ageing, is lacking. Existing, albeit limited data from South America (Brazil and Ecuador), are consistent with the fact that the needs and priorities of older adults may differ according to geographical location [6,7]. With respect to studies directly comparing older adults living in urban and rural locations, only two previous studies have been identified. One of these is a secondary analysis of the Brazil national health survey [8], which reported poorer perception of health in rural compared with urban areas. Interestingly, this contrasted with a cohort study in Ecuador which reported poorer perception of health in older adults in urban compared with rural areas, although this was reversed in the analysis of quality of life [7]. Studies directly comparing the needs and priorities of older people in Brazil across both urban and rural locations are currently lacking. Further data would provide a broader evidence base to inform priorities and policies, tailored for different needs of an extremely diverse population in terms of income, living conditions, educational and cultural backgrounds.

Given this context, this study aimed to identify and analyse the needs and priorities of older people and of stakeholders. The study sought to compare two different gerographical settings in Brazil, one urban and the other rural.

## Methods

The study consisted of two prioritization workshops, in both the urban and rural areas, one for older adults and one for stakeholders. Each workshop was conducted using the Nominal Group Technique (NGT), which we have used previously in a low income country setting [9]. The NGT is a structured procedure for collecting information to determine priorities and/ or problem solve via rapid consensus,. It can be used to explore consumer and/or stakeholder views by encouraging participation, while avoiding problems associated with other group techniques such as dominant personalities [10,11]. Note there is no EQUATOR network guideline for nominal group technique studies.

The workshop locations were within the municipality of Santo André, in the metropolitan region of São Paulo (SP). The estimated population of Santo André is 696,312 in 2023, 19.3% of which (134,388 inhabitants) are ≥60 years old [12]. Santo André faces the demands arising from the significant ageing of its population. In addition, Santo André has a low coverage of the Family Health Strategy, reaching only 25% of the population [13]. The study was performed with older people treated at two Primary Health Units: UBS Vila Guiomar, located in the suburban neighborhood of the city of Santo André and UBS Parque Andreense, located in the Parque Andreense region, located 30 km south of Santo Andre, which is an environmental protection area with rural characteristics.

Full ethical approval for the study was given by Fundação do ABC: FMABC Research Ethics Committee CAAE: 55521022.8.0000.0082xxx and the University of Birmingham Science Technology Engineering and Mathematics Research Ethics Committee ERN_22–0486. Informed consent was obtained from all participants

### Participants

#### Older adults

We aimed to recruit 20 to 30 older people for each workshop. For reasons of feasibility, identification and selection of participants was based on contacts obtained by researchers, i.e., by community health agents or from family health programs.

Inclusion criteria:

People aged 60 years or older residing in urban and rural areas of the city of Santo André SP.

Exclusion criteria:

Any persons aged younger than 60 years of age, older persons who were not residents of the study location, not independently mobile or able to give informed consent.

#### Stakeholders

For this group of participants, we aimed to recruit 20 to 30 stakeholders meeting the inclusion criteria listed below, in both rural and urban locations within Santo André SP. Participants were recruited with the aim of involving all sectors that work with older people in Santo André.

Inclusion criteria:

Stakeholders such as caregivers, senior councils, voluntary and non-governmental organizations, local authorities, community leaders and health and social assistance managers in the urban and rural areas of Santo André SP.

Exclusion criteria:

Any person who was not employed by or involved in an organization that influences or works with the older population.

### Data collection

Each workshop was facilitated by researchers from Centro Universitário Saúde ABC FMABC and Ribeirão Preto Medical School, University of São Paulo. After welcoming the participants to the workshops and offering breakfast, the purpose of the workshop was explained.

An initial plenary discussion was facilitated with all participants to explore the local definition of ageing; this allowed for a shared understanding to frame the workshop in the appropriate context.

Participants were then divided into subgroups of 5 to 7 participants maximum. Subgroups discussed each of the topics listed below for approximately 1 hour, followed by a group-facilitated prioritization exercise (lasting 30–45 minutes), before moving on to the next topic.

Facilitators at each table recorded each group’s suggestions on flipcharts. Discussions at each table were audio recorded in case of later need for clarification. Participants were not asked to identify themselves in the audio recording and no personally identifiable information (including names) were recorded.

During the discussion, the subgroups ranked their top 5 priorities. During the facilitated prioritization exercise, the subgroups shared their ranked priorities with all workshop participants, with the facilitators having the role of encouraging further discussion. After the removal of duplicates by the study team, all participants voted for the selection of the final top 5 priorities by raising their hands. Voting was repeated until 5 priorities were obtained for each topic. Each workshop followed the iterative process described above.

### Topics discussed at the Workshop with older people

1. What is the local definition of ageing? (in plenary only)
2. What is important to you as you get older to ensure you live a healthy and active life?
3. What are the main obstacles (perceived or real) to ensure that you live a healthy and active life?
4. What are the main enablers (perceived or real) to ensure that you live a healthy and active life?

### Topics discussed at the Workshop with stakeholders

1. What is the local definition of ageing? (in plenary only)
2. What is important for the older people in Brazil?
3. What services and family and community structures are available to ensure that the older persons have a healthy and active life in Brazil?
4. What are the main priorities to be addressed in order to keep older people’s health and well-being in Brazil

The questions were constructed for relevance to the respective group and ease of understanding. Our questions sought to identify needs and priorities by eliciting perceptions. In saying this, we specifically asked participants to define ageing rather than their perceptions of ageing, but acknowledge that the definitions received would be based upon perception and personal, cultural and geographical experiences and contexts of ageing. For the stakeholders we separated ‘what is important for older people’ from ‘priorities to be addressed’, not only to directly compare areas of importance identified by older adult/ stakeholder groups, but also to explore whether the obstacles and enablers identified by older people mapped onto priority areas identified by stakeholders. In addition, stakeholders were asked to identify services and family and community structures currently available, to gain insight into how the most important needs of older people were being addressed.

#### Assessment of physical performance

In addition to exploring the needs and priorities of the older people their physical performance was assessed handgrip dynamometry and gait speed using a 4-meter walk. This was done in order to characterise the older group with respect to their functional ability.

Handgrip strength was measured using a manual hydraulic dynamometer (Saehan®, model SH 5001). The test was applied according to the recommendations of the American Society of Hand Therapists [14]. Three measurements of each hand were performed at one-minute intervals. The highest value was used in subsequent analysis [15,16]. Gait speed was measured using the 4-meter walk test at the usual pace, in a clearly demarcated straight path, with an extra space of 1 meter for acceleration and 1 meter for deceleration. Canes and walkers were permitted if the participant normally used this equipment on a daily basis. Two measurements were taken, and the fastest time used in subsequent analysis [17,18].

A data collection sheet was used to capture each participant’s age, sex, and study location (urban or rural), alongside the physical measurements.

### Data analysis

The ranked priorities obtained in response to each question were tabulated prior to translation into English by the study team.

The top ranked responses were categorised using a four-level social-ecological model, which is a framework advanced by the World Health Organisation and which helps to understand the different variables which may influence health and well-being, and how they may interact to determine a community’s health outcomes. Importantly, the model enables stakeholder insights into the variables that influence health and thus may inform interventions to improve health [19]. The levels used in this study include: 1. Individual, which refers to aspects of individuals, for example, their health or physical characteristics; 2. Relationship, referring to relationships with other people, for example family and friends; 3. Environment, which refers to the individual’s living situtation, including its organisations, services, and structures; and 4. Society, which relates to the cultural, political and economic surroundings in which an individual lives [20]. Responses could be categorised into more than one level.

When considering which categorise to place responses under, a deterministic approach was adopted, in which external factors more than free will, determine an individual’s ability to thrive. For example, an individual’s ability to access medical care was considered primarily a factor relating to the societal provision of enablers to access. Two researchers (JD/ CG), categorised the first 30% of responses together; the next 10% of responses were categorised independently until 100% agreement had been achieved; after-which responses were categorised by CG.

Data on handgrip strength and walking speed were calculated for each individual and described for each group of older adults using a measure of central tendency and spread.

## Results

The workshops took place between 21–30 June 2022. Each workshop recruited 15–25 participants (urban older adults n = 17, 15 women; rural older adults, n = 27, 20 women; urban stakeholders n = 20, 18 women; rural stakeholders n = 17, 15 women). See Table 1 for the demographic and physical characteristics of the older adults and Table 2 for the organisations from which stakeholder participants were recruited. Two participants in each older adult group (urban and rural) workshop were found to be <60 y, therefore were asked to not vote.

**Table 1:** Physical characteristics of the older participants.

Area	N	Age	Sex		Walking speed (m/s)	Handgrip Strength (Kg)
		Mean (SD)	Female	Male	Mean (SD)	Mean (SD)
Urban	15	70.1 (6.86)	14	1	1.06 (0.19)	25.50 (7.24)
Rural	25	70.8 (6.53)	18	7	0.94 (0.20)	26.25 (4.77)

**Table 2:** Organisations from which stakeholder participants were recruited.

URBAN stakeholders		RURAL stakeholders	
Professional category	N	Professional category	N
Nurse	6	Community Health Agent	6
Pharmacist	3	Dentist	2
Community Health Agent	2	Dentist assistant	1
Physician	1	Nurse	1
Dietician	1	Nursing technitian	1
Physical Therapist	1	Physician	1
City councilor for health	1	Psychologist	2
Social assistant	1	Technical officer	1
Retired older person	2	Physical Educator	1
Researcher	1	Carer	1
Sanitarian agent	1		

*What is the local definition of ageing*? Table 3 shows the responses to this question. The urban stakeholders top ranked responses broadly focused on ageing in terms of a set of experiences acquired over the years, a phase of life and a natural physiological condition. However, the rural stakeholder top-ranked responses were mainly physiological in nature, defining ageing in terms of a degenerative process and the limitation of physical capacity. The older adults’ responses defined ageing in more positive ways, i.e., ‘changes that we can modulate with physical and mental activities’ (urban) and ‘ageing is a privilege/ ageing is good’ (rural) were the top ranked responses.

**Table 3:** Top-ranking definitions of ageing among older people living in Parque Andreense (rural) and St Andre (urban) locations.

Stakeholders		Older People	
Rural	Urban	Rural	Urban
Heterogenous phenomenon that is person and place dependent	Set of experiences acquired over the years	Ageing is a privilege, the oportunity to learn and live well. Ageing is good and there is no old age	Changes that we can modulate with physical and mental activities
A natural degenerative process	A phase of life with changes and new limitations which are influenced by numerous factors: social, psychological, environment	Natural limitations with advancing age	It is a phase of life that can be good or not, depending on certain factors
Physiological losses equate to senescence; diseases equate to senility	Non-pathological and progressive natural physiological condition	Body changes and adaptation	Feeling good about yourself (the joy of being alive)
Constant mediation between losses and resiliency	Continuation of what the person has always been	Experience lived by each one and interaction with the environment, with limits and possibilities	Continue to live actively and work responsibly
Limitation of physical capacities	The proximity of the end of life, finitude	Acquisition of wisdom and knowledge about life: integration of past, present and future	A phase in which some doors begin to get closed and others become more important: relearning to live with the possibilities that exist

**Fig 1:**
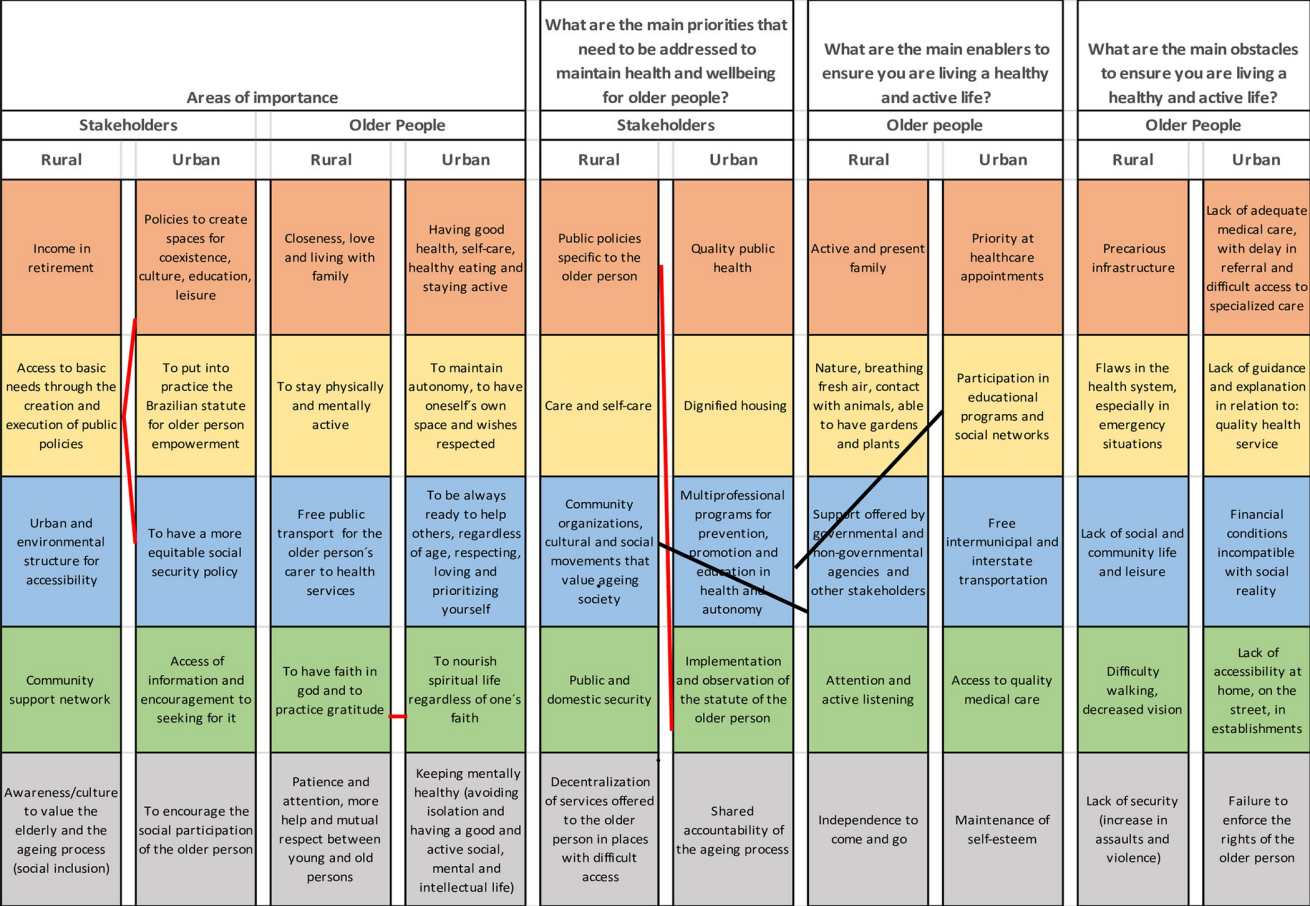
Top-ranked responses from older people and stakeholders living in Parque Andreense (rural) and St Andre (urban) to questions about importance, priorities, obstacles and enablers. Red connecting lines indicating agreeement when comparing within stakeholder and older adult groups and black connecting lines when comparing an older adult enabler to a stakeholder priority.

Fig 1 illustrates the ranked, summarized list of responses to questions about areas of importance, priorities, obstacles, and enablers for older adults and stakeholders in urban and rural areas (see S1 Table for the full ranked list of responses).

*What is important for older people in Brazil*? In response to this question, the top 3 responses from the urban stakeholders related to policies supporting the older person, whereas for the urban older adults, having good health was the highest ranked response. There was no overlap with respect to the top 5 responses between groups (Fig 1). For rural area stakeholders, income in retirement was the highest ranked response whereas for the rural area older adults, closeness, love and living with family was ranked top. Again, there were no shared responses between groups (Fig 1).

Overall, there was very little overlap between stakeholder groups, irrespective of geography, although both groups ranked policies for older people within their top 5. Both urban and rural older adults ranked keeping physically and mentally active, and nurturing spirituality within their top 5 responses (Fig 1).

*What are the main priorities*, *obstacles and enablers for healthy ageing*? The urban stakeholders top ranked priority for healthy ageing was quality public health whereas for the rural stakeholders, it was public policies specific to the older person. The only agreement across the top 5 ranked responses for each stakeholder group was with respect to public policies specific to the older person/ implementation of the statute of the older person.

With respect to obstacles, urban older adults ranked a lack of adequate medical care, including access to specialized care, as their no. 1 obstacle, while rural older adults ranked precarious infrastructure. There was little agreement between older adult groups, with the exception being that that lack of adequate medical care ranked no. 1 by urban older adults was consistent with flaws in the health system, ranked no. 2 by the rural older adults (Fig 1). Comparing obstacles identified by older adults to stakeholder priorities, the urban older adults identified failure to enforce the rights of the older person which mapped onto the stakeholder priority, implementation of the statute of the older person. The rural older adult top-ranked obstacle, precarious infrastructure, mapped onto a stakeholder priority area, decentralization of services in places difficult to access. In addition, public and (lack of) domestic security was also shared between the two groups, ranked 4 and 5 by stakeholders and older adults respectively.

With respect to enablers to healthy ageing, rural older adults ranked active and present family as first, while urban older adults ranked priority at healthcare appointments. There was no overlap between older adult groups. There was little overlap between stakeholder priorities and older adults’ enablers to healthy ageing, with only two responses (i) urban setting; participation in educational programmes and (ii) rural setting; support from key stakeholder groups, mapping an enabler to a priority (Fig 1).

**Fig 2:**
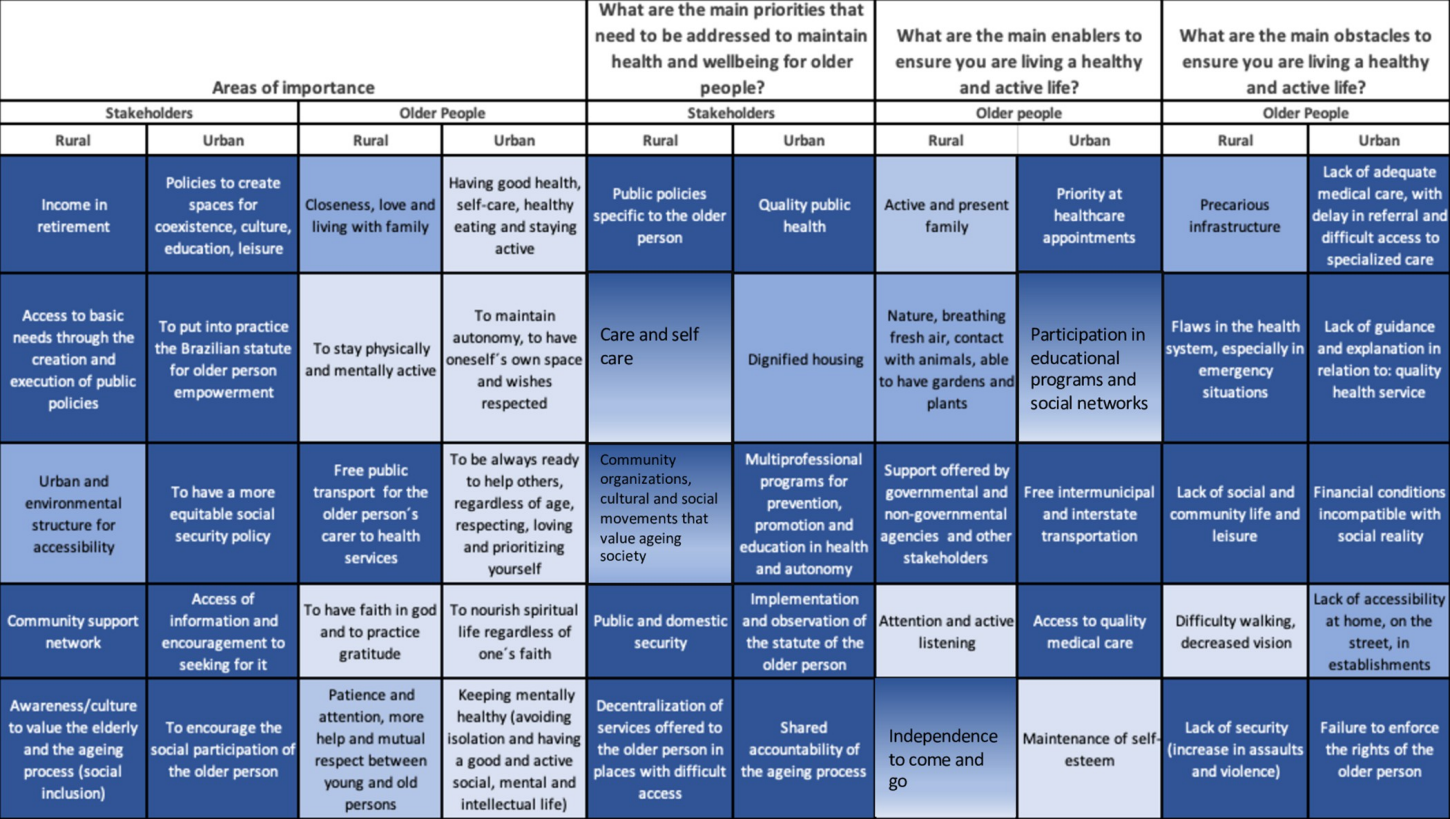
Top ranked responses for stakeholders and older people in St Andre (urban) and Parque Andreense (rural)) categorised using a socio-ecological framework. Note that some responses utilise more than one domain of the social-ecological framework.

Responses categorized using the social-ecological model are shown in Fig 2. When considering what was important for healthy older age, responses from stakeholders in both urban and rural settings were almost all within the Society domain. Only one response from the rural stakeholders was within the Environment domain. The Relationship and Individual domains were not characterised in any stakeholder responses. In terms of priorities (stakeholders), and obstacles and enablers (older adults), urban and rural stakeholder responses were again, mainly within the Society domain, and this was also true for the urban older adults, with a low number of responses within the Environment (n = 1) and Individual (n = 2) domains. The responses of the rural older adults included all domains although most responses were characterised within the Society domain.

The responses to the question ‘what services, family and community structures are available to ensure that older people are able to live healthy active lives in Brazil?’, are illustrated in Table 4.

**Table 4:** Summary of services and family and community structures (see S1 Table) for full list.

What services, and family and community structures are available to ensure that older people are able to live healthy active lives in Brazil?	
Rural stakeholders	Urban stakeholders
Social security; a strong public health system, social services and support; availability of public long-permanence institutions	Legal and social support
Education, culture, leisure and sport opportunities provided by private social entities	Support systems for older people offered by governmental agencies
Guarantee of rights offered by legislation, governmental agencies and public associations	Community activities: religious centers, clubs, residents’ association, seniors’ dance, bingo, bars, fairs, parks, community gardens/community support: parties, visits, services and meetings
Industries, commerce and services	Family ties (i.e. meetings, barbecues, commemorative parties, visits/family structure: care and support for health services (informal care, obligation)
Groups formed by the community itself: participation of religious entities, voluntary support network, university extension projects, NGOs	Actions of social organizations such as the Brazilian Alzheimer´s disease Association, Brasil Parkinson

## Discussion

In this study we sought the opinions of older adults and relevant stakeholders on the definition of healthy ageing in both urban and rural areas in the municipality of Santo Andre, within the metropolitan region of Sao Paulo, Brazil. In addition, we identified commonalities and differences between groups and geographies in response to questions about the most important priorities for healthy ageing, alongside experienced or perceived obstacles and enablers to healthy ageing.

In terms of the local definition of ageing, there was broad concurrence between urban stakeholders and urban older adults (lifelong experiences/ changes which can be modulated). However, the responses of the rural stakeholders contrasted markedly from that of rural older adults. This may have been due to the professional backgrounds of the rural stakeholders, which were less diverse compared with the urban stakeholders, i.e., they were mainly non medical/ medical health professionals, with the predominant group being Community Health Agents (CHAs). Analysis of healthcare in studies focused on Brazilian rural territories identifies the Community Health Agent as the worker who reduces the distance between the population and the health service, in addition to guiding residents who need support for their difficulties on ways to make life easier [21]. Given their role in the community, the definition of ageing was likely to have been driven by their knowledge of the adverse health effects of ageing (i.e., degeneration, physiological loss and limitation). However, this was opposed to the very positive definitions of ageing given by the rural older adults.

With respect to what is important for older adults in Brazil, there was very little concurrence between the ranked responses across both groups and geographical locations. For stakeholders, these differences may have been informed by the different profiles of professional backgrounds between urban and rural settings, with urban being more diverse. However, when the stakeholder group responses were categorized according to the socio-ecological model, they were almost all societal. When the same was applied to the responses of older adults, the predominant categories were individual/ relationships. Although these findings may not be be surprising, they highlight differences in ‘what matters’ between older adults and those who care/ advocate for them and point to potential gaps in communicating priorities between these groups.

There was also relatively little congruence between older adult and stakeholder groups when analysing the priorities of stakeholders in the light of older adults’ experienced or perceived obstacles and enablers to healthy ageing. For example, urban older people identified lack of adequate medical care (delays in referral/ access to specialized care), as their top ranked obstacle while urban stakeholders identified quality public health as a main priority. Similarly, rural older adults identified precarious infrastructure (particularly with respect to transport), as their main obstacle to healthy ageing, and although rural stakeholders ranked public policies for the older person as their main priority, they did not elucidate the areas of focus of such policies. However the urban stakeholders did rank decentralization of servies for older people living in areas difficult to access, as a priority further down the ranking order, perhaps indicating their awareness of the problem.

We highlight in particular, the obstacles expressed by older adults in rural locations which reflect the lack of infrastructure, transport, leisure and cultural opportunities, social assistance, and conditions of access to goods and services. This has been reported in other low and middle income countries; for example in rural Namibia in which environmental barriers to accessing healthcare were experienced by older adults [22]. The lack of distribution of resources reported in the present study was reflected in the rural area older adults top ranked obstacle (precarious infrastructure). It may also explain the importance placed by older adults living in rural areas on closer relationships with the family, plus the provision of social support through different types of services to ensure quality of life.

It is interesting to note that in this study, the rural stakeholders identified a diverse range of available community services and structures across different sectors, but the obstacles identified by rural older adults suggested a lack of effectiveness of such services. Perhaps in recognition of the fact that services and structrues may be available, but not easily accessible, the rural stakeholder group ranked structures for accessibility as being an area of importance. These findings are consistent with a recent study that sought to understand the view of the health-disease-care process presented by older adults 60 years or older, in a rural area of Southern Brazil and which highlighted the need to reorganize health services to achieve a culturally congruent health care model [6]. An earlier study, also of rural area older adults, highlighted the discrepant views of people who lived in rural areas and those of the stakeholders participating in that research [23]. The rural environment was presented as‘invisible’ with respect to social and cultural issues, a finding which is consistent with a previous study in rural areas of Rwanda in which older adults reported feeling neglected by the government [24].

The sparse mapping of older adult enablers to stakeholder priorities was irrespective of geographical location and highlights limited concurrence between the needs and priorities of older people and those who care for them. The voices of stakeholders are louder than older adults, however those voices may not be fully representing the older communities whom they serve. This study thus adds to a very limited literature in this area of research, but highlights the need for more discourse and importantly, inclusion of older adults in priority setting discussions within stakeholder groups.

This study is not without limitations: The rural stakeholder group was constituted mainly by CHAs. A more diverse group may have given different results but CHAs, as previously noted, are often the only contact for many older adults in rural communities. Other stakeholder groups, while representing rural communities, are based within metropolitan areas and this hampers effective communication [21].

There are also considerations relating to the generalizability of our data: The older participants in this study were relatively high functioning, with grip strength comparable to a healthy reference group in the multi-centre Frailty in Brazilian Older People (Fibra BR) study [15] and with faster average walking speeds compared with those of community living older adults participating in the Brazilian Health, Ageing and Wellbeing cohort study [25]. Whether older adults with below average physical function would report similar needs and priorities to the participants of our study, is unclear.

In addition, the Nominal Group Technique, while appropriate as a consensus building methodology for this study, involves roundtable discussion groups, with a recommended maximum of 7 per group [10]. We were therefore limited in terms of total numbers of participants in each workshop as well as the number of workskshops per location in this exploratory study.

The size of Brazil must also be considered: the stakeholder participants of this study found it challenging to capture the services and structures available (although not necessarily accessible) throughout Brazil, perhaps also reflecting geographical differences with respect to the implementation of the National Policy for Older People (PNI), noted in the introduction as not yet being fully applied. In addition, the research reported in this study was based in Sao Paulo state in south-eastern Brazil, where, on the basis of scientific publications, the majority of scientific research is undertaken [26]. Clearly one must be cautious when attempting to generalize the results of this study to the rest of the country in which 20 million adults aged 65 years currently reside.

In conclusion, our study adds to the sparse global literature on the needs and priorities of older adults and our results show the value in opening conversations with older adults about their needs and priorities and for those in advocacy and policymaking roles, to listen in order to focus care and support for older adults, to improve healthy ageing.

## Supporting information

S1 Table Ranked responses from workshops. (PDF)
