# Supplementary material for: Identification of the needs and priorities of older people and stakeholders in rural and urban areas of Santo Andre, Brazil
Source: PLoS One. 2024 May 9;19(5):e0297489. doi: 10.1371/journal.pone.0297489 (PMC11081258; doi:10.1371/journal.pone.0297489)
Supplement: S1 Table — (PDF) [file pone.0297489.s001.pdf]

Supporting Information

S1 Table. Ranked responses from the workshops

S1 Ranked responses from workshops, part 1

	Definition of ageing				What do you think is important for older people in Brazil?			
**Priority Order**	**Rural area stakeholders**	**Urban area stakeholders**	**Rural area older adults**	**Urban area older adults**	**Rural area stakeholders**	**Urban area stakeholders**	**Rural area older adults**	**Urban area older adults**
1	Heterogenous phenomenon that is person and place dependent	Set of experiences acquired over the years	Ageing is a privilege, the opportunity to learn and live well. Ageing is good and there is no old age	Changes that we can modulate with physical and mental activities	Income in retirement	Policies to create spaces for coexistence, culture, education, leisure, from the mapping of places of easy access to the older person, such as health centers, schools, churches, SESAS, residents' association, samba schools, public squares, etc., with more activities for the older person, working on cognitive, mental, emotional stimuli (eg, memory workshops, digital inclusion, opportunities, that is, maintaining the human potential of the older person)/ prevention and health promotion, groups with multidisciplinary teams and health campaigns/tax incentives for the purchase of supplies and materials to carry out therapeutic activities	Closeness, love and living with family	Having good health, self-care, healthy eating and staying active

	Definition of ageing				What do you think is important for older people in Brazil?			
**Priority Order**	**Rural area stakeholders**	**Urban area stakeholders**	**Rural area older adults**	**Urban area older adults**	**Rural area stakeholders**	**Urban area stakeholders**	**Rural area older adults**	**Urban area older adults**
2	A natural degenerative process	it is a phase of life with changes and new limitations which are influenced by numerous factors: social, psychological, environment	Natural limitations with advancing age	It is a phase of life that can be good or not, depending on certain factors	Access to basic needs through the creation and execution of public policies	To put into practice the Brazilian statute of the older person: valorization, elimination of prejudice and production of knowledge. Empowerment of the older person.	To stay physically and mentally active; (i.e. to have a good diet; to avoid addictions; to maintain a good weight; to have social interaction; to have better access to leisure/sports, medication and better medical care)	To maintain autonomy, to have oneself´s own space and wishes respected
3	Physiological losses = senescence; diseases = senility	Non-pathological and progressive natural physiological condition	Body changes and adaptation	Feeling good about yourself (the joy of being alive)	Urban and environmental structure; accessibility	To have a more equitable social security policy (health, social assistance and social security)	Free public transport for the older person´s carer to health services	To be always ready to help others, regardless of age, respecting, loving and prioritizing yourself
4	Constant mediation between losses and resiliency	Continuation of what the person has always been	Experience lived by each one and interaction with the environment, with limits and possibilities	Continue to live actively and work responsibly	Community support network	Access of information and encouragement to seeking for it	To have faith in god and to practice gratitude	To nourish spiritual life regardless of one´s faith

	Definition of ageing				What do you think is important for older people in Brazil?			
**Priority Order**	**Rural area stakeholders**	**Urban area stakeholders**	**Rural area older adults**	**Urban area older adults**	**Rural area stakeholders**	**Urban area stakeholders**	**Rural area older adults**	**Urban area older adults**
5	Limitation of physical capacities	The proximity of the end of life, finitude	Acquisition of wisdom and knowledge about life: integration of past, present and future	A phase in which some doors begin to get closed and others become more important: relearning to live with the possibilities that exist	Awareness/education/culture to value the elderly and the ageing process (social inclusion)	To encourage the social participation of the older person in conferences to guarantee their rights and value. Reconstruction of social parameters, review of the organization of society to start changes in public policies	To have patience and attention with the older person, more help and mutual respect between young and old persons	Keeping mentally healthy (avoiding isolation and having a good and active social, mental and intellectual life).
6	Social and cultural phenomenon that devalues the person	Mental process that can be positive or negative	Biological process of every human being, in which you make choices, but are not well valued	The accumulation of experiences with attitudes		To work on concepts of citizenship from the ground up and place politicians with a better social vision on aging	To perform physical exercise. Investment in adequate urban structure, equipment for muscle strengthening. More security to come and go	To want to live, to be happy and have peace of mind
7	Privilege, but with unadmitted losses	An heterogeneous, plural phenomenon		Radical change in life for which we are not prepared		To discuss and reflect on the responsibilities delegated to the older person, as they are mothers, grandmothers and women with physical, mental and social overload	Faster referral to specialists in order to prevent the aggravation of diseases	To have a physically active life
8	To remain independent despite getting frail	The sum of a few (many) years of life		Physical limitation and loss of strength and memory		To invest in public and community security, integrated with other public policies due to violence, especially in the periphery, including mental problems, alcoholism and drugs	Self-worth, self-recognition as a priority	To have good financial conditions

	Definition of ageing				What do you think is important for older people in Brazil?			
**Priority Order**	**Rural area stakeholders**	**Urban area stakeholders**	**Rural area older adults**	**Urban area older adults**	**Rural area stakeholders**	**Urban area stakeholders**	**Rural area older adults**	**Urban area older adults**
9	Acquisition/development of wisdom	The possibility of living longer with health, independence and autonomy		Ageing is awful		To improve education by ensuring more access and opportunities	Knowledge of diseases for prevention and self-care	
10	isolation/depression/retirement	The use of a vital capacity, which someone is born with					To live one day at a time; positive thinking; leaving the bad things behinds and to overcome obstacles	
Un-ranked		a moment of reflection of the lived experiences					Access to informatic technologies and encourage the use of apps (CESA could offer it; public politic)	
							To valuate the knowledge of young people such as in the use of new technologies	

File S1 Ranked responses from workshops, part 2

	What services, and family and community structures are available to ensure that older people are able to live healthy active lives in Brazil?		What are the main obstacles to ensure that you live a healthy and active life?		What are the main priorities that need to be addressed to maintain health and wellbeing for older people in Brazil?		What are the main enablers to ensure you are living a healthy and active life?	
**Priority Order**	**Rural area stakeholders**	**Urban area stakeholders**	**Rural area older adults**	**Urban area older adults**	**Rural area stakeholders**	**Urban area stakeholders**	**Rural area older adults**	**Urban area older adults**
1	Social security, INSS (national institute for social security): retirement, pension, aid in case of disease. SUS: integral attention to health. SUAS: senior card, Brazil-aid, viva-leite, interaction services, Long-permanence institutions.	services with legal support: statute for the older person, police station for the older person, prosecutor's office for the older person, council for the older person, commission for the older person at Brazilian Lawyers Assocaition, dial 100	Precarious infrastructure without sidewalks, without footbridges, leading to reduced mobility; precarious public transport; lack of street signs and radars; bad roads	Lack of adequate medical care, with delay in referral and difficult access to specialized care	Public policies specific to the older person, including listening, guidance, awareness of families and the older person, with equity and guarantee of rights	Quality public health	Active and present family	Priority at healthcare appointments

	What services, and family and community structures are available to ensure that older people are able to live healthy active lives in Brazil?		What are the main obstacles to ensure that you live a healthy and active life?		What are the main priorities that need to be addressed to maintain health and wellbeing for older people in Brazil?		What are the main enablers to ensure you are living a healthy and active life?	
**Priority Order**	**Rural area stakeholders**	**Urban area stakeholders**	**Rural area older adults**	**Urban area older adults**	**Rural area stakeholders**	**Urban area stakeholders**	**Rural area older adults**	**Urban area older adults**
2	Education: EJA, CESA, culture/leisure/sport. S System (SENAI, SESC, SESI, SENAC)	government agencies: CRISAS, reference centers, URSI, PAI, sports centers, NASFs, NASI, day care, good food, green currency, free transport, discount for culture, police station for the elderly, CRAS, CREAS/ multidisciplinary groups in UBS, multiprofessional clinics , CISI, CER, elderly-friendly hospitals, elderly-friendly city, mature city (Paraíba), Vila Dignity (SP), Vive Mais Program (Paraná), Public and Private LTCFs, Specialty Center for the Elderly, IPGG, Hospital do Amor palliative care, outdoor gym, college for the elderly, program "são paulo friend of the elderly"	Flaws in the health system, especially in emergency situations	Lack of guidance and explanation in relation to: quality health services, prescriptions, exams and consultations, transport and rights of the older person	Care and self-care in its entirety (physical, social, emotional, spiritual and financial) for healthy aging	Dignified housing	In this specific place, nature, breathing fresh air, contact with animals and being able to have gardens and plants	To learn and put into practice manual, mental and occupational activities, in addition to exchanging experiences and skills + staying active through manual, intellectual and voluntary work, maintaining life goals, a routine and participating in educational programs and social networks

	What services, and family and community structures are available to ensure that older people are able to live healthy active lives in Brazil?		What are the main obstacles to ensure that you live a healthy and active life?		What are the main priorities that need to be addressed to maintain health and wellbeing for older people in Brazil?		What are the main enablers to ensure you are living a healthy and active life?	
**Priority Order**	**Rural area stakeholders**	**Urban area stakeholders**	**Rural area older adults**	**Urban area older adults**	**Rural area stakeholders**	**Urban area stakeholders**	**Rural area older adults**	**Urban area older adults**
3	Rights guarantee system: statute of the older person, legal councils, public defensory, police station for the older person, Brazilian Lawyers Association (Commission for the Older Person)	community activities: religious centers, clubs, residents' association, seniors' dance, bingo, bars, fairs, parks, SESCs, community gardens/community support: parties, visits, services and meetings	Lack of social and community life and leisure, which leads to isolation/sadness/ depression/distres s	Financial conditions incompatible with social reality	Community organizations, cultural and social movements to value ageing	Multiprofessional programs for prevention, promotion and education in health, aiming at preserving autonomy and independence	Support offered by governmental and non-governmental agencies and other stakeholders (primary and family healthcare units , including community health agents; well-being group (physical activity, auriculotherapy, handcraft); garbage collection, family club (NGO), small businesses, sub-prefecture that helps in the orientation of construction and renovations, Education for the young and aged person).	Free intermunicipal and interstate transportation
4	Industries, commerce and services	Family ties (i.e. meetings, barbecues, commemorative parties, visits/family structure: care and support for health services (informal care, obligation)	Difficulty walking, decreased vision	Lack of accessibility at home, on the street, in establishments	Public and domestic security	Implementation and observation of the statute of the older person	Attention and active listening	Access to quality medical care

	What services, and family and community structures are available to ensure that older people are able to live healthy active lives in Brazil?		What are the main obstacles to ensure that you live a healthy and active life?		What are the main priorities that need to be addressed to maintain health and wellbeing for older people in Brazil?		What are the main enablers to ensure you are living a healthy and active life?	
**Priority Order**	**Rural area stakeholders**	**Urban area stakeholders**	**Rural area older adults**	**Urban area older adults**	**Rural area stakeholders**	**Urban area stakeholders**	**Rural area older adults**	**Urban area older adults**
5	Groups formed by the community itself: participation of religious entities, voluntary support network, university extension projects, NGOs	actions of social organizations: Brazilian Alzheimer´s disease Association, Brasil parkinson	Lack of security (increase in assaults and violence)	Failure to enforce the rights of the older person (inadequate sidewalks, inadequate public transport, etc.)	Decentralization of services offered to the older person in places with difficult access	Strengthening of public policies aimed at the older person	Independence to come and go	Maintenance of self-esteem
6		private initiatives: condominiums, walks in the city center, trips to the supermarket, hairdressing salon, neighbors, relationship APPs, travel, fishing, pet and plants	Insufficient retirement income, reducing the quality of life	Lack of adequate spaces for the older person to do physical activities (water aerobics). Lack of courses for the older person (languages, computers, dance, painting, etc.)		Consolidation of the empowerment of the older person/family in society	Small community where everyone knows each other, organization	To keep an active social life
7			Lack of attention and empathy, lack of communication	Lack of job opportunity/prejudice		Training of multidisciplinary professionals who deal with the older person	Having more time for self-care, freedom to do what one wants whenever one wants	Priority parking
8			Lack of running water (one goes for days without water), lack of sewage	Lack of acceptance of this new phase of life by the older persons themselves		Basic and decent income	Home appliances (washing machine, refrigerator, microwave, radio, car, cell phone, etc.)	To have good nutrition and regular practice of exercises

	What services, and family and community structures are available to ensure that older people are able to live healthy active lives in Brazil?		What are the main obstacles to ensure that you live a healthy and active life?		What are the main priorities that need to be addressed to maintain health and wellbeing for older people in Brazil?		What are the main enablers to ensure you are living a healthy and active life?	
**Priority Order**	**Rural area stakeholders**	**Urban area stakeholders**	**Rural area older adults**	**Urban area older adults**	**Rural area stakeholders**	**Urban area stakeholders**	**Rural area older adults**	**Urban area older adults**
9			Older persons being carers of older persons	Limitations that the family imposes such as lack of respect for the autonomy and will of the older person		Accessibility (adequate sidewalks)	Use of walking devices (cane, guava tree stick)	To be in good financial condition to ensure a good quality of life
10			Overload of responsibilities with grandchildren, children and aggregates	Not being able to count on help from family members and other people (friends and neighbors)		Shared accountability of the ageing process	Pay half-price in cinemas and theaters	The experiences acquired over time
Unranked			Lack of motivation					Dismissal of the necessity of physical presence for proof of life for older people with limited mobility
								To have warmth and support from family, friends and neighbors + live in harmony within marriage, home and family
								Easy and consigned loan for older people
